# Dental Implant Macro-Morphology and Surface Characteristics: A Narrative Review

**DOI:** 10.7759/cureus.106541

**Published:** 2026-04-06

**Authors:** Pierluigi Valente, Lapo Sbrenna, Andrea Mascolo, Andrea Sbrenna, Francesco Valente

**Affiliations:** 1 School of Dentistry, Vita-Salute San Raffaele University, Milan, ITA; 2 School of Dentistry, Alma Mater Studiorum University of Bologna, Bologna, ITA; 3 Medical Education and Simulation, European Institute for Medical Studies, St. Julian's, MLT; 4 Dentistry, Humanis Dental Center, Perugia, ITA; 5 Oral Implantology, San Damiano Dental Clinic, Rome, ITA

**Keywords:** bone–implant contact (bic), dental implants, implant design, implant macrogeometry, implant macro-morphology, implant surface, marginal bone level (mbl), osseointegration, primary stability, surface roughness

## Abstract

The biological and mechanical stability of dental implants is closely influenced by their macro-morphology, micro- and nano-scale surface topography, and physicochemical surface properties, all of which modulate cell adhesion, osteoblastic activity, and osseointegration. An integrated understanding of the interactions between implant design, surface treatments, and the peri-implant biological response is essential for optimizing clinical outcomes and reducing the risk of complications. This narrative review critically examines the role of implant macro-geometry and the principal surface modification strategies, with particular focus on sandblasted, large-grit, acid-etched (SLA), anodized, nanostructured, and titanium plasma-sprayed (TPS) surfaces, as well as thread geometry and surface roughness.

SLA surfaces are widely considered among the most established and extensively investigated implant surface treatments and have been consistently associated with high survival rates and favorable marginal bone level (MBL) stability. Anodized and nanostructured surfaces may offer additional potential benefits in terms of biofunctionalization and antimicrobial activity; however, some evidence suggests slightly lower cumulative survival rates (CSR) and MBL outcomes. Implant macro-geometry, including body shape and thread configuration, plays a critical role in load distribution, primary stability, and bone-to-implant contact (BIC).

Overall, available evidence suggests that implant success results from the synergistic interplay between macro-geometry, surface characteristics, and implant-abutment connection design, rather than from a single variable. Moderately rough surfaces combined with optimized implant design appear to represent a well-balanced approach in terms of osseointegration, mechanical stability, and biological safety. Further studies are warranted to better define the optimal parameters of surface roughness, chemical composition, and implant design, and to improve long-term clinical predictability.

## Introduction and background

Modern dental implantology is based on a fundamental concept: the biological and mechanical stability of an implant is closely influenced by its macro- and micro-scale morphology, as well as by the physicochemical properties of its surface. Titanium and its alloys are widely recognized as the materials of choice due to their excellent biocompatibility, corrosion resistance, and favorable mechanical properties [1]. The implant surface represents the first point of contact with the alveolar bone and plays a critical role in osseointegration, modulation of the peri-implant cellular response, and achieving primary stability. Recent nano-scale surface modifications have been shown to influence protein adsorption, cell adhesion, and peri-implant osteogenesis, although the long-term clinical benefits remain to be fully established [2,3].

Factors such as macro-, micro-, and nano-scale topography, surface morphology, chemical composition, electrical charge, and hydrophilic or hydrophobic properties are known to influence osteoblast adhesion, proliferation, and bone tissue maturation [4]. A surface characterized by optimized micro- and nano-scale topography, when appropriately designed, may promote osteoblastic differentiation and increase bone-to-implant contact (BIC), with potentially beneficial effects on bone regeneration and primary stability. The osseointegration process follows a well-defined sequence, beginning with the formation of woven bone, followed by lamellar bone, and continuous remodeling of the bone-implant interface over time [5]. A key clinical challenge lies in balancing surface characteristics to maximize osseointegration while minimizing biological complications. While moderately rough surfaces have been shown to enhance BIC and accelerate osseointegration, increased surface roughness may also favor bacterial adhesion and biofilm formation, potentially increasing the risk of peri-implant inflammation and disease.

Primary implant stability depends on multiple factors, including implant body design, diameter, length, and thread geometry [6]. Specific thread parameters, such as pitch, depth, and helix angle, have been shown to influence stress distribution and mechanical stability, particularly in low-density bone [7]. Bone density at the recipient site and the chosen surgical technique also affect insertion torque. Experimental studies suggest that primary stability is significantly influenced by the combined effects of implant shape, dimensions, and surface topography, as well as by surgical technique and bone density at the recipient site. In particular, tapered implants generally require higher insertion torque compared to cylindrical designs, while surface treatments, such as acid-etched or anodized, may enhance initial stability [8,9].

Despite the large body of available research, universally accepted guidelines for defining optimal implant surface topography are still lacking. Manufacturers propose surfaces with varying characteristics, and reported clinical outcomes show variability depending on both surface treatment and implant design. The present review aims to critically analyze the influence of implant macro-geometry and surface characteristics on the peri-implant biological response and clinical outcomes.

Aim of the review

This narrative review aims to provide a comprehensive overview of implant macro-morphology and surface modification strategies, with particular emphasis on their biological and mechanical implications. Specifically, this review seeks to critically evaluate how implant design and surface characteristics influence osseointegration, peri-implant tissue response, and clinical outcomes. By integrating evidence from in vitro, in vivo, and clinical studies, the objective is to clarify the potential role of these factors in optimizing implant performance and long-term predictability.

## Review

Search strategy

The literature included in this narrative review was identified through searches conducted in PubMed and Scopus databases up to January 2026, without predefined time restrictions. The search strategy included combinations of keywords and Boolean operators such as “dental implants” AND “surface roughness” AND “osseointegration,” as well as terms related to implant macro-geometry, surface topography, surface treatments, and peri-implant tissue response. Studies were selected based on their conceptual relevance to the biological and mechanical implications of implant design and surface characteristics, with particular attention to clinical outcomes, experimental evidence, and translational applicability. Priority was given to peer-reviewed articles, including clinical studies, as well as relevant in vitro and animal research that provide mechanistic insights.

As a narrative review, this study does not follow the Preferred Reporting Items for Systematic Reviews and Meta-Analyses (PRISMA) guidelines and does not include a formal risk-of-bias assessment. No strict inclusion or exclusion criteria were predefined, consistent with the narrative nature of the review. While this approach represents an inherent methodological limitation, it allows for a broader synthesis and conceptual interpretation of the interactions between implant macro-geometry, surface properties, and peri-implant biology.

Discussion

Surface treatments of dental implants represent a key factor in modulating the biological response at the bone-implant interface. Through controlled modifications of micro- and nano-scale topography, as well as physicochemical surface properties, it is possible to significantly influence cell adhesion, osteoblastic differentiation, and osseointegration processes [10]. Over time, several surface treatment strategies have been developed with the aim of optimizing the peri-implant biological response and improving both short- and long-term clinical outcomes.

This section provides a critical analysis of the studies selected for this review, examining the main types of implant surfaces currently used in clinical practice. Particular attention is given to both the underlying biological mechanisms and the available evidence in terms of implant survival, marginal bone level (MBL) stability, and peri-implant tissue behavior, in order to better define the role of different surface treatment strategies in contemporary clinical practice. Overall, available evidence suggests that no single surface treatment alone determines clinical success; rather, outcomes are influenced by the complex interaction between surface characteristics, implant design, and host-related factors.

Sandblasted, Large-Grit, Acid-Etched (SLA) Surfaces

The combination of mechanical sandblasting and chemical acid etching represents the most established method for achieving controlled surface micro-roughness. Sandblasting, typically performed using alumina or titanium dioxide particles, produces a uniform macroscopic roughness, while acid etching generates a micro-topography characterized by peaks and valleys. SLA surfaces significantly increase initial BIC, promoting osteoblast adhesion and proliferation, as well as pro-angiogenic macrophage activation. A mean surface roughness (Ra) in the range of 1.0-2.0 μm appears to optimize osteogenic stimulation without significantly increasing titanium debris release.

Long-term clinical studies suggest slightly higher survival rates and reduced MBL compared to anodized or untreated surfaces, supporting SLA as a reference technique for accelerating osseointegration [5]. In particular, a recent meta-analysis including clinical studies with follow-up of up to 10 years compared SLA and anodized implants, reporting a cumulative survival rate (CSR) of 99% and MBL of 0.69 mm for SLA implants, versus a CSR of 98% and MBL of 1.02 mm for anodized implants [11], further supporting the long-term stability of SLA surfaces. A recent study comparing three implant systems made of commercially pure titanium and titanium alloys subjected to anodization or sandblasting and acid etching reported significant differences in surface roughness and composition. Particle abrasion followed by acid etching was shown to be effective on titanium alloys as well, confirming the versatility of this treatment across different implant materials [12].

Anodization and Nanopatterning

Electrochemical anodization produces nanoporous surfaces with nanostructures that may enhance cell adhesion, osteoblastic differentiation, and biofunctionalization, while also modulating the immune response and potentially conferring antimicrobial properties. More advanced surface engineering techniques combine micro- and nano-topographies with bioactive coatings, creating multi-scale surfaces capable of simultaneously improving osseointegration and antibacterial activity [13]. Susin et al. compared anodized abutments with standard machined titanium abutments in a mini-pig model. No significant differences were observed in terms of inflammation, epithelial attachment length, mucosal height, or bone density. However, at six weeks, crestal bone levels were significantly more stable around anodized abutments (P = 0.046), suggesting a potential advantage during early soft tissue healing [14]. From a biological perspective, such graded surfaces may provide site-specific optimization of the bone-implant interface. Smoother or minimally rough regions at the collar level may contribute to reduced plaque accumulation and improved soft tissue integration, whereas moderately rough and porous regions toward the apex may enhance osteoblast adhesion, bone ingrowth, and mechanical interlocking.

This spatial modulation of surface characteristics may therefore support both peri-implant tissue stability and primary stability. Emerging in vitro evidence supports this concept. A recent 3D cell culture study by Ayyildiz et al. reported increased expression of osteogenic differentiation markers in implants with gradually varying anodized surface topographies compared to non-graded surfaces, although no significant differences in cell viability or proliferation were observed [15].

These findings suggest that graded surface designs may influence osteogenic signaling pathways, potentially enhancing early biological responses at the bone-implant interface. However, the current evidence remains limited and is largely based on experimental models. The extent to which these biological advantages translate into improved clinical outcomes, such as enhanced osseointegration, reduced marginal bone loss, or increased long-term implant survival, remains to be clarified. Further well-designed clinical studies are therefore required to validate the potential benefits of graded surface topographies in implant dentistry.

Titanium Plasma Spray (TPS) and Advanced 3D Target-Ion-Induced Plasma Sputtering (TIPS) Surfaces

Titanium plasma spray (TPS) consists of the deposition of molten titanium droplets, creating macro- and micro-rough surfaces with cavities and niches that promote bone ingrowth and direct BIC. However, excessively rough surfaces may increase the risk of long-term failure, and potential coating delamination and particle release represent relevant clinical limitations [16]. Preclinical studies on 3D-printed titanium implants have introduced advanced surface treatments such as target-ion-induced plasma sputtering (TIPS), which uses plasma-generated ions to create controlled micro- and nano-roughness.

This technique increases the bone-implant contact area and enhances osteoblast adhesion. In animal models, 3D implants treated with TIPS showed significantly higher BIC and initial bone density compared to untreated or conventionally SLA-treated implants [17]. It should be noted that a substantial portion of the available evidence is derived from in vitro and animal studies, and therefore may not fully reflect clinical performance in human subjects. While these findings are promising, the evidence is currently limited to preclinical models and requires further clinical validation.

Zirconia Implant Surfaces

Zirconia implant surfaces have gained increasing interest as a potential alternative to titanium, particularly due to their aesthetic properties and possible advantages in soft tissue biocompatibility. However, the available evidence regarding osseointegration and bone-implant interaction remains limited and heterogeneous. In a systematic review with meta-analysis, Hafezeqoran et al. compared BIC and removal torque (RT) values between titanium and zirconia implants with different surface modifications [18]. No significant differences in BIC were observed between titanium and machined zirconia implants, whereas acid-etched zirconia implants showed significantly higher BIC values. Non-modified zirconia implants also demonstrated favorable BIC values compared to some modified surfaces. As with titanium implants, surface modifications appear to influence both the rate and quality of osseointegration, with high BIC values observed in experimental models [5].

More recent studies highlight the role of specific surface treatments in optimizing zirconia osseointegration. Jin et al. reviewed commercially available zirconia implants and reported that subtractive protocols-such as sandblasting, acid etching, laser etching, and combinations thereof-modulate surface roughness, wettability, and osteogenic signaling. Laser-modified surfaces exhibited the highest BIC and osteopontin expression, along with favorable hydrophilicity and minimal microcrack formation. Combined treatments, including sandblasting followed by laser or acid etching with post-milling heat treatment, showed similarly favorable biological responses [19]. Advanced coating strategies have also been explored. Huang et al. investigated plasma-sprayed nanostructured zirconia (NSZ) coatings on titanium implants in a rabbit model. NSZ-coated implants displayed higher surface roughness and wettability compared to titanium, resulting in enhanced early-stage bone formation, higher BIC, and more condensed trabecular bone during the first weeks of healing [20].

Recent clinical evidence, including a systematic review and meta-analysis of one-piece zirconia implants, suggests that these implants achieve osseointegration comparable to titanium, with stable marginal bone levels over short-, mid-, and long-term follow-up, and a low risk of implant fracture. Evidence for two-piece zirconia implants remains scarce [21]. These findings suggest that bioactive zirconia coatings can further enhance early osseointegration beyond conventional surface modifications. Overall, the available evidence indicates that zirconia, particularly when appropriately surface-treated or coated, may achieve effective osseointegration comparable to titanium. However, the heterogeneity of data and the limited number of long-term clinical studies currently restrict direct comparisons and definitive conclusions.

Implant Macro-Morphology

Implant macro-morphology includes body shape (cylindrical, tapered, or hybrid), length, diameter, thread pitch, and thread angle. These parameters influence load distribution, BIC, and primary stability. A reduced thread pitch increases the bone-implant contact area, enhancing primary stability in low-density bone (type IV), whereas a wider pitch facilitates insertion and distributes loads over a larger surface area. Thread angle modulates load transmission: sharper angles may reduce cortical stress and minimize microtrauma, while wider angles may improve resistance to displacement under immediate loading conditions. Implant body design also affects geometric adaptation to the bone site: tapered implants promote bone self-compression and primary stability in low-density bone, whereas cylindrical implants may offer greater predictability in dense bone.

In vitro studies, biomechanical simulations, and clinical evidence suggest that the combined effect of thread pitch, thread angle, and implant body shape plays a critical role in primary stability and load distribution, particularly in type IV bone [22]. A recent meta-analysis of randomized clinical trials indicates that implant length is not a decisive macro‑morphological factor for clinical success, as extra-short implants (≤6 mm) achieved survival outcomes comparable to longer implants [23]. These findings highlight the importance of tailoring implant macro-design to site-specific bone conditions in order to optimize primary stability and potentially improve long-term outcomes.

Biological and Clinical Implications

The combination of macro-geometry, micro-topography, and nano-topography, together with optimized physicochemical surface properties, allows effective modulation of the peri-implant biological response, promoting osseointegration, osteoblast adhesion and proliferation, and potentially antimicrobial activity [14,24,25]. Integration with a stable implant-abutment connection and appropriate transmucosal materials contributes to reducing micromovements, preserving the mucosal barrier, and supporting long-term stability [17,26]. Collectively, these factors contribute to maximizing the biofunctionalization of the implant-prosthetic system and supporting long-term clinical success [14,27]. The presence and release of titanium particles represent an additional relevant factor in modulating the peri-implant biological response.

Available evidence suggests that particle release may begin during implant insertion and continue over time under functional loading, as well as following professional maintenance and decontamination procedures [28]. Particle size appears to play a critical role: in particular, titanium dioxide (TiO₂) nanoparticles, particularly in the anatase crystalline form, which is characterized by higher surface reactivity, have been associated with potential pro-inflammatory effects at the local level. Experimental studies further suggest that such particles may be phagocytosed and, in animal models, translocate to systemic sites. However, the actual clinical impact of these phenomena remains debated and requires further investigation [29].

Long-term clinical evidence confirms the high predictability of the main implant surface types. Wennerberg et al. evaluated implants with different surfaces (turned, TPS, blasted, anodized, blasted and acid-etched) with follow-up ≥10 years, reporting survival rates ranging from 82.9% to 100%, with mean marginal bone loss below 2 mm across all surfaces [27]. Moderately rough surfaces showed higher survival compared to smoother or excessively rough surfaces; anodized surfaces exhibited the lowest probability of failure, whereas more aggressive TPS surfaces were associated with increased risk. These findings suggest that, despite technical differences, the main implant surfaces provide high success rates when combined with proper surgical execution and appropriate prosthetic management.

Additional systematic evidence supports the clinical reliability of sandblasted and acid-etched surfaces, also in immediate or early loading protocols. Chambrone et al. reported survival rates of approximately 95% for SLA implants and up to 97% for highly hydrophilic modified surfaces (SLActive), with no significant differences compared to delayed loading protocols [30]. More recent systematic evidence suggests that implant material characteristics and surface coatings may also influence peri-implant inflammatory response. In a PRISMA-based systematic review, Matvijenko et al. reported statistically significant differences in marginal bone loss (MBL) associated with certain surface modification techniques, such as fluoride treatment and sandblasted acid-etched surfaces, whereas other coating materials and implant alloys did not show consistent effects on bleeding on probing or probing depth [31].

In addition to hard tissue integration, increasing attention has been directed toward the role of implant-abutment surface characteristics in promoting soft tissue attachment. Surface modifications specifically designed to enhance soft tissue integration, such as those based on tailored surface chemistry and topography of transmucosal components, may contribute to improved mucosal sealing and peri-implant tissue stability, potentially reducing bacterial infiltration and protecting the underlying bone [32]. In this context, emerging technologies, including artificial intelligence and advanced biomaterials, are expected to further influence implant dentistry, although robust clinical evidence is still limited [33]. 

Although multiple studies have investigated implant surface characteristics, the variability in study design and clinical context indicates that these features likely function as modulators of biological risk rather than as independent predictors of clinical outcomes. While increased surface roughness enhances osteoblastic activity and BIC, it may also facilitate bacterial adhesion and biofilm accumulation, potentially increasing the risk of peri-implant inflammation and disease. Therefore, an optimal balance between biological integration and microbial susceptibility is essential in implant surface design.

The main implant surface types, their macro- and microstructural features, biological effects, and reported clinical outcomes are summarized in Table 1.

**Table 1:** Dental implant surface characteristics, macro-morphology, and clinical/biological outcomes Data derived from previously published studies [2-5,11-17,18-25,28-32] SLA: sandblasted, large-grit, acid-etched; Ra: surface roughness; BIC: bone-to-implant contact; CSR: cumulative survival rate; MBL: marginal bone loss; TPS: titanium plasma spray; TIPS: target-ion-induced plasma sputtering; Ti: titanium

Implant surface/feature	Micro-/nano-topography	Macro-morphology	Biological effects	Clinical outcomes	Limitations/notes
SLA	Micro-rough (Ra 1–2 µm), some nano-features [5,30]	Cylindrical/tapered	↑ Osteoblast adhesion, ↑ BIC, pro-angiogenic [12,31]	High CSR (99%), MBL ~0.69 mm [11,30]	Slight titanium particle release [28]
Anodized	Nanoporous, controlled oxide layer	Cylindrical/tapered	↑ Biofunctionalization, potential antimicrobial effect [13,14,15,32]	CSR slightly lower (98%), MBL ~1 mm [11]	Limited long-term data
TPS	Macro- + micro-rough	Cylindrical/tapered	↑ Bone ingrowth [16]	Good early osseointegration	Risk of delamination, debris release, and very rough surfaces may increase long-term failure [16,29]
3D-TIPS/advanced 3D surfaces	Controlled micro- + nano-roughness	Custom 3D-printed	↑ Osteoblast adhesion, ↑ BIC [17]	Preclinical: faster osseointegration [17]	Clinical data limited
Zirconia	Smooth to micro-rough (acid-etched variants) [19]	Cylindrical/tapered	Biocompatible, favorable soft tissue response [19]	BIC comparable to Ti if acid-etched [5,18,19,20,21]	Limited long-term clinical evidence
Thread design/macro-geometry	Pitch, angle, body shape [22]	Conical, cylindrical, hybrid	↑ Primary stability, load distribution [14,24,25]	Important for type IV bone, short implants [22,23]	Optimized in combination with surface [2,3,4]

## Conclusions

Sandblasted and acid-etched surfaces remain the clinical standard for predictable osseointegration. Anodized and nanostructured surfaces may offer additional biofunctional and antimicrobial advantages, while graded or multi-scale designs show promise for optimizing site-specific interactions at both hard and soft tissue levels. Overall, implant success appears to depend on the synergistic interplay between macro-geometry, surface characteristics, implant-abutment connection design, and host-related factors, rather than on any single parameter. Surface characteristics may therefore act as modifiers of biological risk rather than independent determinants of clinical outcomes. Further well-designed clinical studies are needed to clarify the long-term benefits and clinical relevance of advanced surface technologies.
